# Fyn, an important molecule in the brain, is a potential therapeutic target for brain tumours

**DOI:** 10.3389/fphar.2024.1485919

**Published:** 2024-12-04

**Authors:** Chongxi Xu, Yi Tang, Xing Lu, Ruiqi Chen

**Affiliations:** ^1^ Department of Neurosurgery, West China Hospital, Sichuan University, Chengdu, China; ^2^ Department of Pharmacy, Sichuan Clinical Research Center for Cancer, Sichuan Cancer Center, Sichuan Cancer Hospital & Institute, Affiliated Cancer Hospital of University of Electronic Science and Technology of China, Chengdu, China; ^3^ Department of Gynecological Nursing, West China Second Hospital, Sichuan University, Chengdu, China

**Keywords:** Fyn, brain tumours, Src family of kinases, gliomas, therapeutic target

## Abstract

Under normal physiological conditions, Fyn, a nonreceptor tyrosine kinase, is involved in signal transduction pathways in the nervous system and in the formation and activation of T lymphocytes. Fyn is a member of the Src family of kinases (SFKs) and plays a role in cell morphogenic transformation, motility, proliferation, and death, which in turn influences the development and progression of various cancer types. SFKs are overexpressed or hyperactive in tumours, and they are engaged in several signalling pathways that lead to tumour development. Inhibition of Fyn can enhance patient outcomes and prolong survival. Thus, Fyn is a desirable therapeutic target in a variety of tumour types. To lay the groundwork for further investigation and targeted therapy in tumours, in this article, we review the most recent findings on the function of Fyn in tumours, with an emphasis on its role in gliomas. Understanding the function of Fyn during tumourigenesis and development and in resistance to anticancer therapeutic agents can aid in the development and application of innovative medicines that specifically target this kinase, thus improving the management of cancers.

## Introduction

Among the earliest kinases to be identified were the Src family of kinases (SFKs) (Frame, 2002), which contains 11 members, of which 8—c-Src, Fyn, Yes, Lck, Lyn, Hck, Fgr, and Blk—have been extensively researched. While Fyn, Yes, and c-Src are expressed throughout the human body, Blk, Hck, and Fgr are exclusively expressed in certain tissues (Thomas and Brugge, 1997). Among the SFKs, c-Src has been the most extensively researched in terms of cancer biology, as it is a crucial molecule in the genesis, progression, and resistance of tumours to treatment (Larsen et al., 2015). Over the past 10 years, it has become increasingly clear how other members of the SFK family—including Fyn—are involved in different facets of cancer biology.

Myristic and palmitic acids bind and localize Fyn to the inner layer of the cell membrane (Alland et al., 1994). Like other SFKs, Fyn function is controlled by tyrosine phosphorylation- and dephosphorylation-induced intermolecular interactions. Many target proteins, such as focal adhesion kinase (FAK) and breast cancer anti-oestrogen resistance protein 1 (BCAR1), undergo tyrosine phosphorylation as a result of Fyn activation (Yeatman, 2004). Fyn also regulates cell growth, survival, adhesion, cytoskeletal remodelling, motility, axon guidance, synaptic function, myelination in the central nervous system, platelet activation, and T-cell receptor signalling, among other various biochemical processes (Kinsey, 2014). The function of Fyn in the brain is discussed specifically in this review, particularly in relation to several elements of the pathophysiology of brain tumours, such as gliomas.

## Gene and protein structure

Chromosome 6q21 contains genetic information for Fyn, a 59-kDa protein with 537 amino acids (p59-FYN, Slk, Syn, MGC45350, Gene ID 2534). Fyn belongs to the Src family and was first discovered in 1986 (reported as Syn or Slk) using probes constructed from v-yes and v-fgr (Semba et al., 1986; Alland et al., 1994). Fyn is mostly found in the cytoplasmic leaflet of the plasma membrane, where it phosphorylates tyrosine residues on important targets connected to a wide range of signalling pathways.

**FIGURE 1:**
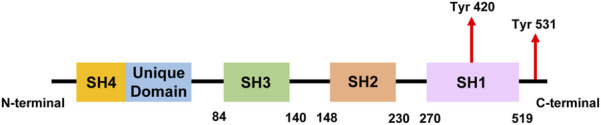
A simplified linear structure of Fyn. The numbers indicate amino acid residues and the locations of tyrosine residues that can be phosphorylated during Fyn activation.

Three distinct transcript isoforms of Fyn have been discovered, and among the three genomic sequences, isoform 1 (isoform a, Fyn [B]) is the longest and was the first to be discovered. Compared with isoform 1, isoform 2 (also known as isoform b, Fyn [T]) is more capable of mobilizing cytoplasmic calcium and is more likely to be expressed in T cells (Thomas and Brugge, 1997). Some of the variations in the regulation of these two isoforms can be attributed to alterations in the linker region between the SH2 and SH1 domains (Alland et al., 1994). In the area close to the start of the kinase domain and the end of the SH2 domain, isoforms 2 and 1 differ by approximately 50 amino acids (Figure 1). While most tissues express a combination of both isoforms (Thomas and Brugge, 1997), the brain expresses high levels of Fyn(B), whereas T cells highly express Fyn(T). Isoform 3 (isoform c) lacks exon 7 (FynD7) and has been reported to be expressed in blood cells; however, no translated protein has been identified (Goldsmith et al., 2002). Other transcript variants have also been identified, but they have not yet been linked to any disease state.

Fyn regulates the phosphorylation of intracellular tyrosine proteins by interacting with numerous cell surface receptors, such as those on mast cells and T cells (Davidson et al., 1994). The development of oligodendrocytes, keratinocytes, and natural killer cells is a result of the physiological function of Fyn in cellular processes such as lymphocyte receptor signalling, and Fyn is known to be involved in adhesion, cell migration, and platelet activation (Cary et al., 1996; Zamoyska et al., 2003; Reddy et al., 2008).

## Fyn in the brain

Fyn plays a significant role in both adult brain function and brain development. The biological roles of Fyn in the brain have been thoroughly studied using transgenic animal models. These studies revealed that Fyn is an essential component for the growth and operation of the central nervous system. Mice lacking Fyn exhibit a variety of brain abnormalities, such as distorted hippocampal architecture and aberrant long-term potentiation (Kojima et al., 1997), impaired spatial learning and increased sensitivity to ethanol (Grant et al., 1992), which indicates the physiological importance of Fyn in a variety of brain communication pathways. Furthermore, Fyn has been detected in several brain regions, such as glial cells in white matter tracts and cultured oligodendrocytes, and plays significant roles in CNS myelination, which is the process by which a myelin sheath forms around a nerve fibre. Actually, myelination occurs when Fyn is most active in the brain (Osterhout et al., 1999). Sperber and colleagues reported that Fyn knockout (KO) mice exhibit substantial myelin loss in the forebrain at all ages (from 14 days to 1 year). Their investigation demonstrated that Fyn has a distinct function in myelination according to the number of oligodendrocytes and myelinated fibres, and their findings were supported by the results of experiments in which an inactivated form of Fyn (containing a single amino acid substitution) was tested (Sperber et al., 2001).

Fyn plays a role in the morphological differentiation that causes oligodendrocytes to generate neurites, which are projections that extend from the cell body of a neuron. Fyn interacts with α-tubulin, a tubulin family member that plays a crucial role in the polar orientation and nucleation of microtubules, which are cytoskeletal structures necessary for the generation of neurites. More specifically, membrane-associated α-tubulin combines with Fyn to form a complex that participates in the signalling pathway that initiates the nucleation of membrane-associated microtubules. In one study, pretreatment of P19 embryonal cancer cells with wortmannin or SFK inhibitors consistently inhibited α-tubulin complex nucleation activity (Macurek et al., 2008). Additionally, the association of Fyn with Tau, a cytoskeletal protein that binds to tubulin to stabilize microtubules in the brain, promotes this process (Lee et al., 1998).

Additional research revealed that Tau–Fyn interactions in oligodendrocytes are important for developmental myelination and that some human CNS neurodegenerative diseases, such as multiple sclerosis, may be caused by dysfunctional Tau–Fyn interactions. For example, in multiple sclerosis, axonal degeneration is the primary cause of clinical decline (Belkadi and LoPresti, 2008).

Interestingly, tyrosine-phosphorylation of Tau has also been reported in human prostate cancer cells (Sangrajrang et al., 1998), and it was previously reported that tyrosine within Tau can be phosphorylated *in vitro* by the oncogene v-fms, a Src family tyrosine kinase expressed in human histiocytic lymphoma cells (Kim et al., 1991). Since cancer and oncogenes are associated with abnormal cell proliferation, these findings in nonneuronal cells may serve to link the tyrosine phosphorylation of Tau to cell signalling pathways that induce cell growth. Preclinical studies in Fyn-deficient mice have shown that Fyn plays a role in development (Grant et al., 1992; Lowell and Soriano, 1996). Fyn also participates in the cell cycle (Yasunaga et al., 1996; Sette et al., 2002). Previous studies have demonstrated the upregulation of Fyn in the AD brain (Shirazi and Wood, 1993) and the presence of a Fyn phosphorylation site in Tau(Lee et al., 2004), which further supports a role for Fyn in the pathogenesis of neurodegenerative disorders.

Some studies have focused on the nonreceptor tyrosine kinase Fyn, which interacts with polyproline helices in Tau through its SH3 domain (Lee et al., 1998), and interestingly, also regulates seizure susceptibility (Cain et al., 1995; Kojima et al., 1998). Tau reduction is protective in a Fyn-dependent model of AD (Roberson et al., 2011). Thus, studies have shown that Tau targets Fyn to dendrites, where Fyn facilitates N-methyl-d-aspartate (NMDA) receptor-mediated dysfunction and aberrant Tau phosphorylation (Ittner et al., 2010; Larson et al., 2012).

The role of Tau or Fyn, or both, as part of a shared mechanism of neuronal hyperexcitability and seizures has been reported (Roberson et al., 2011; Putra et al., 2020). Genetically ablated Fyn or pharmacological inhibition of Fyn/SFK prevents neuronal loss and decreases network hyperexcitability and seizure progression (Kaufman et al., 2015; Sharma et al., 2018). The proconvulsive property of Fyn is also mediated through microglia, independently of Tau, and Fyn/SFK inhibition significantly decreases epileptogenesis (Sharma et al., 2021). These findings suggest the role of Fyn and Tau in promoting seizures and epilepsy. A recent study in an experimental temporal lobe epilepsy (TLE) model demonstrated that amyloidogenic pathways promote Tau pathology during early epileptogenesis (Thom et al., 2011). Similarly, another group reported the deposition of Aβ in the hippocampus of human patients with chronic TLE (Gourmaud et al., 2020), which suggests possible early interactions between Fyn-Tau complexes and amyloid pathways during epileptogenesis that may persist in the chronic stage of epilepsy. Moreover, the degree of Fyn-Tau interactions was shown to be positively correlated with NR2B-PSD95 complexes, Tau phosphorylation and increased Fyn levels in the human epileptic brain. The binding of Fyn-Tau is also correlated with microgliosis, which suggests its contribution to the neuroinflammatory state of the human epileptic brain (Putra et al., 2024). Recently, tat-Tau PxxP5/6, a peptide that targets Fyn-Tau interactions, was shown to prevent Aβ-induced neurotoxicity *in vitro*, but the efficacy of this peptide has not been tested *in vivo* (Rush et al., 2020; Roth et al., 2024). Thus, blocking Fyn and Tau interactions with this peptide inhibitor could further validate the outcomes of pharmacological inhibition of Fyn/SFK in an epilepsy model and could identify the Fyn-Tau interaction as a potential therapeutic target in epilepsy.

In the healthy brain, the microglial response is protective and may decrease once damage has occurred; however, under pathological conditions, microglia become activated and release reactive oxygen species (ROS), nitric oxide (NO) and proinflammatory cytokines, such as tumour necrosis factor-α (TNF-α), interleukin (IL)-1β (IL-1β) and interferon gamma (IFN-γ) (Yan et al., 2014). These products further accelerate microglial activation by binding to their microglial cell surface receptors to sustain chronic inflammation (Kim and de Vellis, 2005). Fyn kinase has been demonstrated to be involved in this process through its role in microglial activation via the Fyn‒PKCδ signalling axis and NOD-like receptor protein 3 (NLRP3) inflammasome. Under pathological conditions, such as those observed in neurodegenerative diseases, these proinflammatory cytokines bind to their receptors on the microglial cell surface, which leads to further propagation of aberrant microglial activation (Xu et al., 2021). Studies involving cell culture and transgenic Fyn^−/−^ mice have consistently demonstrated that Fyn is required for cytokine release and activation of iNOS(Ko et al., 2018; Sharma et al., 2018). More recently, Fyn was shown to be critical for the upregulation and posttranslational modification of Kv1.3, a voltage-gated calcium channel, in microglia (Sarkar et al., 2020). Kv1.3 may play a key role in sustaining the chronic neuroinflammatory response observed in PD.

In addition to microglia, Fyn is also expressed to a lesser extent in astrocytes, where it has a key role in astrocytic migration in response to neuronal signals (Dey et al., 2008). Astrocytes are also involved in the regulation of the CNS immune response, and similarly to microglia, they play both beneficial and detrimental roles in the brain’s response to insult or injury (Giovannoni and Quintana, 2020). Fyn kinase, particularly the isoform FynT, plays a role in the astrocyte-mediated production of proinflammatory cytokines (IL-1β and IL-6) via the PKCδ signalling axis, and additionally, Fyn kinase inhibition attenuates this response (Lee et al., 2017). This response is associated specifically with chronic exposure to inflammation, which suggests the involvement of astrocytes in a more chronic insult. Fyn also appears to play a regulatory role in the astrocytic expression of iNOS following inflammatory stimulation, as increased iNOS expression is observed in Fyn-deficient astrocytes (Ko et al., 2018). Interestingly, this seems to contrast with findings in Fyn-deficient microglia, in which iNOS expression is decreased (Panicker et al., 2015). These data suggest that Fyn kinase is not only involved in the upregulation of the microglia-mediated release of iNOS but may also be involved in the downregulation of the astrocyte response.

The postsynaptic density (PSD), the primary cytoskeletal specialization at neuronal excitatory synapses, is where PSD95, NMDAR, and AMPAR, among other proteins, reside. Fyn and other SFK members are involved in synaptic transmission and plasticity at excitatory synapses. PSD95 plays a crucial role in the multiprotein complex formed by NMDARs by directly attaching to the NR2 subunit of NMDARs. Additionally, PSD95 interacts with the SH2 domain of Fyn, and it has been suggested that this interaction helps Fyn phosphorylate tyrosine residues within the NMDAR subunit NR2A (Tezuka et al., 1999; Kalia and Salter, 2003). Thus, Fyn (as well as Src) controls the phosphorylation of the NMDAR complex and increases NMDAR activity, which results in the generation of NMDAR-dependent synaptic potentiation (Salter and Kalia, 2004).

**FIGURE 2:**
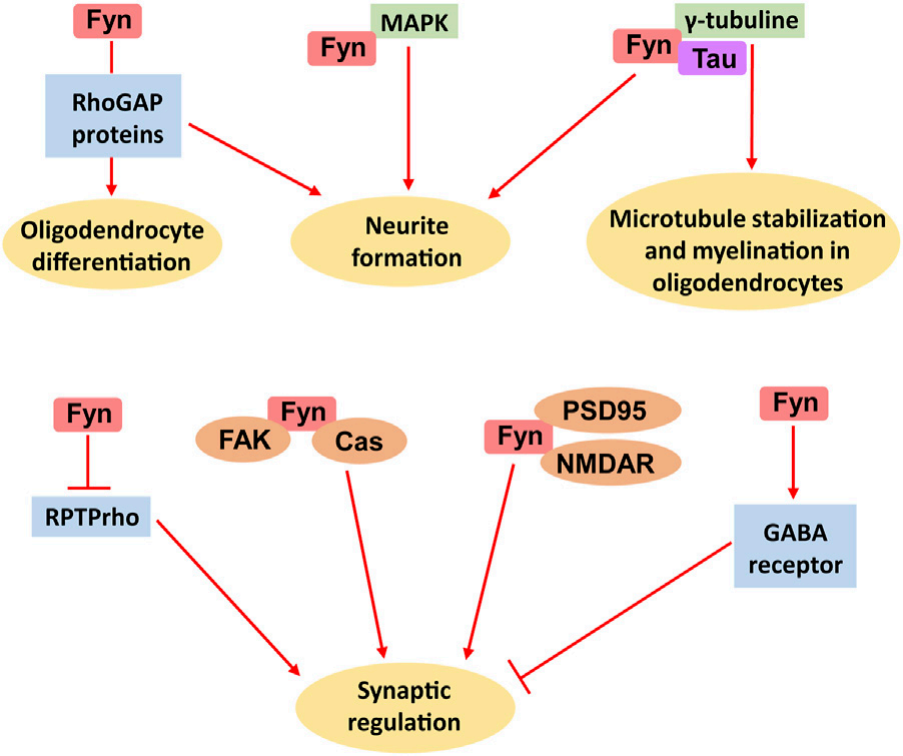
Multiple biological functions of Fyn in the brain. The main roles of Fyn include oligodendrocyte differentiation, neurite formation, microtubule stabilization, myelination by oligodendrocytes and synaptic regulation.

Additionally, Fyn modulates other CNS signalling proteins; Fyn works in tandem with Cas and other FAK family kinases to control the shape of dendritic spines, which are the primary sites of the postsynaptic components of excitatory synapses in the mammalian central nervous system (Bourgin et al., 2007) (Figure 2).

## Biological functions of Fyn in cancer

### Fyn regulates the tumour cell cycle

As a member of the Src family of kinases, Fyn uses anti-SH2 to halt cytoplasmic division following mitosis, which inhibits cell division (Tominaga et al., 2000; Ng et al., 2005). Fyn controls the stabilization and polymerization of microtubules, which in turn controls the development of mitotic spindles. Through enhanced microtubule aggregation, Fyn stimulates the development of mitotic spindles, which accelerates the progression of M phase (Okamoto et al., 2016). Insufficient Fyn activity results in cytoplasmic division failure and prevents the occurrence of mitosis (Levi et al., 2010). In addition, depending on its N-terminal length, Fyn can be confined to the cortical membrane-bound region during cytoplasmic division (Czech and Vander Zanden, 1991). It is believed that cortical Fyn regulates cytoplasmic division (Levi et al., 2010). According to the findings above, Fyn prevents pericellular growth and suppresses mitotic progression.

### Fyn regulates tumour cell adhesion

Dasatinib-induced Fyn inhibition or Fyn silencing has been shown to improve intercellular adhesion (Fenton et al., 2015). T-cell activation is followed by initial T-cell adhesion, which occurs independently of Fyn kinase activity. Nevertheless, noncatalytically functioning Fyn is necessary for late cell attachment (Chapman et al., 2012). In integrin α6-deficient acute lymphoblastic leukaemia (ALL), phosphorylated Fyn (pTyr530) is increased and mediates the development of chemoresistance through adhesion (Gang et al., 2020).

### Fyn regulates tumour cell proliferation

Fyn is a proto-oncogene and member of the Src family. Numerous studies have shown that Fyn inhibits apoptosis and promotes cancer cell growth. As an essential mediator and regulator of mitogenic signalling, Fyn controls cell entry into the cell cycle, growth, and proliferation (Gururajan et al., 2015). Fyn is expressed more frequently in thyroid cancer at both the mRNA and protein levels, thus facilitating cell division and preventing apoptosis (Zheng et al., 2017). MicroRNA-125a-3p directly targets Fyn to inhibit both its expression and activity; additionally, this RNA drives the production of Fyn downstream proteins and cell cycle arrest, which further suppresses cell proliferation. This implies that Fyn stimulates tumour cell growth (Ninio-Many et al., 2013). Increased Fyn expression and activity in chronic granulocytic leukaemia facilitates the transition from chronic to acute disease and increases the rate of cell division (Singh et al., 2012). Osteoclast apoptosis is inhibited by Fyn, thus resulting in osteoclast growth (Kim et al., 2010). Samples from patients with acute myeloid leukaemia (AML) exhibit dysregulated Fyn expression, which is linked to both oncogenic FLT3-ITD and wild-type FLT3. The SH2 structural domain of Fyn and the kinase activity of FLT3 are required for this association. FLT3 contains several Fyn binding sites, and Fyn expression increases STAT5 phosphorylation and colony formation but also marginally increases the phosphorylation of AKT, ERK1/2, and p38. Furthermore, a poorer prognosis in AML patients is associated with increased Fyn expression in conjunction with the FLT3-ITD mutation, which is enriched in the STAT5 signalling pathway. These findings show that Fyn preferentially activates the STAT5 pathway in conjunction with the oncogenic FLT3-ITD gene during cell transformation to increase AML cell proliferation (Chougule et al., 2016). By upregulating Fyn expression and downregulating miR-153-3p expression, LINC00152 promotes the growth of oesophageal squamous cell carcinoma (ESCC) (Liu D. et al., 2019). Fyn phosphorylates PIKE-A in glioblastoma, which encourages the binding of this protein to AMPK, reduces the ability of AMPK to prevent tumour growth, and increases the rate at which tumour cells proliferate (Zhang et al., 2016). The proliferation of pancreatic cancer cells is hindered by the inhibition of Fyn activity (Je et al., 2014). Increased Fyn activity in skin squamous cell carcinoma (SCC) cells decreases Notch1/NICD mRNA and protein expression levels and stimulates STAT3 phosphorylation to promote tumourigenesis and proliferation (Zhao et al., 2009). By inhibiting cellular senescence and promoting the formation of malignant gliomas, Fyn phosphorylates STAT3 and increases G6PD expression (Sun et al., 2021). Fyn also interacts with ARHGEF16 to stimulate the growth of colon cancer cells (Yu B. et al., 2020). Moreover, melanoma cell proliferation is inhibited by the Fyn/STAT3 pathway (Tang et al., 2020). Through the activation of GluN2b and the control of the AKT protein kinase signalling pathway, Fyn promotes the growth of pancreatic cancer (Dong et al., 2020).

### Fyn and the immune response

Increased expression or activation of Src and its downstream protein PI3K enhances the growth and activation of lymphocytes, macrophages, dendritic cells, and natural killer (NK) cells. Fyn splice variation was originally observed in T lymphocytes (Sugie et al., 2004; Abram and Lowell, 2008). According to one study, Fyn activity is necessary for antigen-specific T-cell activation, as the inhibition of Fyn activity significantly reduces the T-cell response (Sugie et al., 2004). Dasatinib, a Bcr-Abl tyrosine kinase inhibitor that also inhibits SFKs, has been used to treat CML patients in clinical trials. This medication causes transitory immunosuppression, which is characterized by the activation of T lymphocytes and haemophilic cells by T-cell receptors and IgE(Sillaber et al., 2009). The effects of SFK inhibitors on patients treated with dasatinib were described in a previous study. Lipid disturbance and a lack of Fyn binding to intraluminal leaflets reduce NK cell activation (Wu et al., 2021). FasL overexpression increases the death of NK cells and T cells by attracting Fyn via proline-rich domains (Malarkannan, 2020).

Elevated Fyn expression in glioma cells diminishes the immunological response against glioma, whereas Fyn inhibition enhances the effectiveness of antiglioma immunotherapy (Comba et al., 2020). Cytokine production in NK and T cells is selectively regulated by the Fyn-ADAP pathway (Gerbec et al., 2015). ADAP, SKAP55, and SHP-2 are directly bound and phosphorylated by Fyn, while SHP-2 interacts with PD-1 to promote PD-1+ CTLA-4+ CD8^+^ TILs in malignancies (Li et al., 2015).

### Fyn in tumour drug resistance

Numerous studies have revealed that Fyn promotes drug resistance in tumours, which is a significant obstacle to the successful treatment of cancer patients. The susceptibility of TKI-resistant cells to the dual BCR-ABL1/Src inhibitor dasatinib increases when Fyn protein expression levels are knocked down versus when Fyn activity is inhibited (Irwin et al., 2015). In one study, the knockdown of Fyn kinase via pharmacological inhibition or siRNA resensitized a BCR-ABL inhibitor imatinib-resistant chronic granulocytic leukaemia (CML) cell line (IM-R cells) to imatinib (Fenouille et al., 2010). Moreover, the susceptibility of tamoxifen-sensitive cells to tamoxifen therapy decreased after Fyn overexpression. Moreover, tamoxifen sensitivity was restored upon the suppression of Fyn expression, and mechanistic research revealed that Fyn counteracts the antiproliferative effects of tamoxifen by activating crucial cell cycle-related proteins (Elias et al., 2015). Compared with control cells, BC cells are more chemosensitive to DOX when miR-381 downregulates Fyn, which deactivates MAPK signalling (Mi et al., 2018). Drug resistance has been shown to develop in dasatinib-resistant cells through the overactivation of Fyn (Airiau et al., 2017). Tamoxifen resistance in breast cancer (ER^+^) is caused by Fyn, and the proliferation of tamoxifen-resistant cells and the correlation of tamoxifen-resistant cells with a poor prognosis in breast cancer are markedly decreased by the use of a Fyn inhibitor or by the knockdown of Fyn expression (Joshi et al., 2016). Fyn plays a role in anticancer drug resistance; in K562 cells, increased Fyn expression was linked to imatinib resistance (Grosso et al., 2009). The imatinib resistance observed in prostate cancer patients is modulated by Fyn via its interaction with miR-128/193a-5p/494 (Ergün et al., 2023). Fyn is therefore strongly expressed in numerous types of cancer-resistant cells and contributes to the emergence of treatment resistance in cancer.

### Glioma

Gliomas, which account for 40% of all primary brain tumours, are the most common type of brain cancer. The term glioma is used to characterize all primary brain cancers that involve central nervous system (CNS) glial cells (Liang et al., 2020). According to the IARC’s GLOBOCAN report on cancer incidence and mortality, 308,102 new cases of central nervous system malignancies were diagnosed worldwide in 2020, which accounted for 251,329 deaths (Bray et al., 2024). An imbalance between cell proliferation and apoptosis, with reduced apoptosis due to the overexpression of antiapoptotic genes in cells, and increased malignant proliferation resulting in tumour development, is the current theory of glioma development; however, its exact pathogenesis is unknown (Poonan et al., 2021). Fyn tyrosine kinase is overexpressed in human gliomas and is a downstream target of the oncogenic receptor tyrosine kinase pathway (Comba et al., 2020), where abnormal SFK activation results in numerous protumor consequences, such as decreased apoptosis, increased angiogenesis, and enhanced cell invasion, motility, and proliferation (Eskilsson et al., 2016). In patients with GBM, Src and Fyn, two downstream targets of the EGFR oncogenic signalling pathway, are often overexpressed. Since glioblastoma activates the EGFR signalling pathway along with Fyn and Src, blocking Fyn and Src may increase the effectiveness of anti-EGFR-targeted therapy (Ahluwalia et al., 2010). Many malignancies have EGFR mutations. However, EGFR inhibitor-induced clinical responses are rare and fleeting. Fyn and Src were identified as putative EGFR effectors in early studies. Moreover, molecular circuits connecting EGFR/EGFRVIII to Fyn and Src have been shown to increase glioblastoma invasion and tumour growth in a variety of cell lines and mouse models. These findings in tumour tissues validate the clinical significance of the abovementioned results, as glioblastoma patients with activated EGFR signalling also often exhibit activated Fyn and Src. These findings indicate that Fyn and Src are clinically significant targets and that blocking them could improve the effectiveness of treatments that target EGFR (Lu et al., 2009).

Fyn establishes a complex regulatory pathway involving specific molecules during glioma development. T-cell immunoglobulin and mucin domain containing-3 (Tim-3), which is highly expressed in gliomas, is a typical immune checkpoint molecule (Kim et al., 2020). Galectin-9 (Gal-9) is the primary ligand that activates Tim-3. According to one study, Tim-3 interacts with Fyn kinase and binds Gal-9 (Wolf et al., 2020). A member of the PIKE family, PtdIns-3-kinase enhancer-activating Akt (PIKE-A), is an oncogenic factor that is essential for the survival and proliferation of cancer cells (Zhang et al., 2019). Numerous investigations have demonstrated that PIKE-A expression is elevated in a variety of malignancies, including glioblastoma, and that it facilitates the growth, invasion, and survival of glioblastoma cells in situations of cellular energy stress (Jia et al., 2016). Fyn can phosphorylate the GTPase PIKE-A, which prevents its degradation (Zhang et al., 2016). Interestingly, PIKE-A increases the growth of glioblastoma and suppresses cellular senescence by triggering the Fyn-mediated STAT3 signalling pathway, which increases the activation of the pentose phosphate pathway (PPP), promotes G6PD expression, and increases DNA synthesis and ROS detoxification (Sun et al., 2021). G6PD is essential for cancer progression, but its underlying mechanisms are still unknown. Some researchers have demonstrated that Fyn directly phosphorylates and increases G6PD activity in response to EGFR activation, which then activates the PPP. Furthermore, Fyn expression, malignancy, and prognosis are correlated with G6PD pY481 in human glioblastoma. These results demonstrate a critical function for Fyn-dependent G6PD phosphorylation in tumour development stimulated by EGF (Liu et al., 2019b).

A recent article reported that the Fyn gene, together with other genes involved in brain development and neural differentiation, is strongly enriched in astrocytoma, a common and lethal human malignancy (Wu et al., 2010). Moreover, Fyn and c-Src are effectors of oncogenic EGFR signalling in glioblastoma and enhance invasion and tumour cell survival *in vivo*. In one study, the pan-SFK inhibitor dasatinib consistently inhibited invasion, promoted tumour regression, and induced apoptosis *in vivo*, which significantly prolonged the survival of mice in an orthotopic glioblastoma model. This study demonstrated a mechanism linking EGFR signalling with Fyn and Src activation to promote tumour progression and invasion and provided a rationale for combined anti-EGFR and anti-SFK targeted therapies (Lu et al., 2009). In addition, a phosphotyrosine proteomic screen identified novel signalling molecules, including JAK1, STAT1, cortactin, FER, p130Cas, c-Src and Fyn, as molecules that undergo tyrosine phosphorylation and activation in human malignant mesothelioma. They also confirmed that known signal transduction pathways previously implicated in mesothelioma, such as EGFR and Met, are coactivated in most human mesothelioma specimens and tested cell lines. Since all these enzymes seem to be hyperactivated in malignant mesothelioma cell lines, dual or multitargeted inhibition of some of these kinases is likely to be more efficacious than inhibition of a single tyrosine kinase to prevent potential antiproliferative activity in glioma treatment (Menges et al., 2010).

Finally, cognitive impairments and recurring seizures affect up to 80% of all patients with diffuse glioma and up to 50% of patients with glioblastoma multiforme (GBM) during the course of the disease (van Breemen et al., 2007; van Kessel et al., 2017). Although no single experimental model recapitulates the full diversity of human gliomas, insight into the emergence of hyperexcitability and the natural history of epileptogenesis in cortical networks, along with the opportunity to link these to specific oncogenic drivers (Yu K. et al., 2020), can provide a precise, mechanism-based approach to individualized medical management of this serious tumour comorbidity. The mechanisms underlying peritumoral hyperexcitability in glioma are likely reciprocal in that greater excitability drives tumour progression, and greater tumour progression promotes further hyperexcitability (Hatcher et al., 2020). These pathological waves involve a massive intracellular calcium influx mediated in part by NMDA receptor activation, which transiently silences neuronal activity and briefly impairs the precise coding of high-frequency synaptic inputs in recovering neurons (Revah et al., 2019). Thus, along with the loss of peritumoral synaptic inhibition, increased extracellular glutamate due to the overexpression of the glial glutamate antiporter system xc-has been proposed to be an important contributor to epileptogenesis in tumour-related epilepsy (Robert et al., 2015; Sørensen et al., 2018). Fyn can regulate neuronal activity, and Tau interacts with Fyn via its amino-terminal projection domain (Lee et al., 1998). Fyn phosphorylates NMDA receptor subunit 2 to facilitate interaction of the NMDA receptor complex with PSD-95 (Nakazawa et al., 2001; Rong et al., 2001), which links NMDA receptors to synaptic excitotoxic downstream signalling (Salter and Kalia, 2004). Disruption of the NMDA receptor/PSD-95 interaction prevents excitotoxic damage in cultured neurons and in a rat model of stroke without affecting synaptic NMDA currents (Aarts et al., 2002), which may decrease neuronal activity and inhibit tumour progression.

## Conclusion

Src is a well-known oncogene, but its family members, such as Fyn, have received less attention even though they may be more crucial in some malignancies than c-Src. Since Fyn participates in multiple intracellular signalling pathways to govern processes such as cell proliferation and differentiation, interest in Fyn has increased nearly a century after it was first described. New research has demonstrated that Fyn is aberrantly and extensively expressed in a variety of cell types. Apart from its direct contribution to the control of signalling pathways, Fyn is also linked to certain signalling molecules that are specific to tumour cells. These molecules collectively contribute to the advancement of cancer metastasis and growth. In addition, numerous highly selective Fyn/Src inhibitors have been synthesized and shown to be successful in clinical studies. For example, saracatinib is a highly specific small molecule inhibitor of the SRC family of kinases with an IC50 value of 10 nm against Fyn. In a phase II clinical trial, saracatinib was confirmed to act as a metastasis suppressor in prostate cancer in the initial stages (Posadas et al., 2016). Saracatinib can be used alone or in combination with radiotherapy to treat malignant tumours, such as glioblastoma (Yun et al., 2021). Dasatinib is a novel and effective multitargeted inhibitor of kinases of the SRC family, as well as several other kinases. In a phase II clinical trial in patients with melanoma, dasatinib was not significantly effective because of poor patient tolerance and dosage reductions (Kluger et al., 2011). Ine one study, immunotherapy plus dasatinib treatment in mice with liver metastases from colorectal cancer significantly increased immune cell infiltration into the tumour, thereby enhancing antitumour immunity (Kadota et al., 2022). Chemotherapy combined with dasatinib is also significantly more effective in the treatment of tumours than chemotherapy alone (Ma et al., 2019; Wang et al., 2022). However, Fyn is still a difficult target. However, whether Fyn promotes malignancy in all tumour types is unclear. Due to its strong similarities with other Src family kinases and its widespread expression throughout the body, targeted therapy may have unanticipated and unwanted off-target consequences. To improve the prognosis of cancer patients, more research is necessary to understand the activation and inactivation of Fyn as well as its mode of action in other cancers.
